# Occurrence and Characterization of Methicillin Resistant *Staphylococcus aureus* in Processed Raw Foods and Ready-to-Eat Foods in an Urban Setting of a Developing Country

**DOI:** 10.3389/fmicb.2019.00503

**Published:** 2019-03-14

**Authors:** Mohammad Aminul Islam, Sahana Parveen, Mahdia Rahman, Mohsina Huq, Ashikun Nabi, Zahed Uddin Mahmood Khan, Niyaz Ahmed, Jaap A. Wagenaar

**Affiliations:** ^1^Food Microbiology Laboratory, Laboratory Sciences and Services Division, International Centre for Diarrhoeal Disease Research, Bangladesh, Dhaka, Bangladesh; ^2^Institute of Food Science and Technology, Bangladesh Council of Scientific and Industrial Research, Dhaka, Bangladesh; ^3^Department of Botany, Jahangirnagar University, Dhaka, Bangladesh; ^4^Laboratory Sciences and Services Division, International Centre for Diarrhoeal Disease Research, Bangladesh, Dhaka, Bangladesh; ^5^Department of Infectious Diseases and Immunology, Utrecht University, Utrecht, Netherlands; ^6^Wageningen Bioveterinary Research, Lelystad, Netherlands

**Keywords:** methicillin resistant *S. aureus*, raw meat, ready-to-eat foods, MLST, *spa* typing

## Abstract

Infections by methicillin-resistant *Staphylococcus aureus* (MRSA) are gradually increasing in the community. In this study, we investigated a total of 162 food samples including 112 ready-to-eat (RTE) foods and 40 processed raw meat and fish samples collected from retail vendors in Dhaka, Bangladesh and determined the occurrence of toxigenic *S. aureus* and MRSA. Around 22% of samples were positive for *S. aureus*, RTE foods being more positive (23%) than the processed raw meat/fish samples (18%). Among 35 *S. aureus* isolates, 74% were resistant to erythromycin, 49% to ciprofloxacin and around 30% to oxacillin and cefoxitin. Around 37% of isolates were resistant to ≥3 classes of antibiotics and 26% of isolates (*n* = 9) were identified as MRSA. Majority of the isolates were positive for enterotoxin genes (74%), followed by *pvl* gene (71%), toxic shock syndrome toxin (*tsst*) gene (17%) and exfoliative toxin genes (11%). Multi locus sequence typing (MLST) of 9 MRSA isolates identified four different types such as ST80 (*n* = 3), ST6 (*n* = 2), ST239 (*n* = 2) and ST361 (*n* = 2). *spa* typing of MRSA isolates revealed seven different types including t1198 (*n* = 2), t315 (*n* = 2), t037 (*n* = 1), t275 (*n* = 1), t304 (*n* = 1), t8731 (*n* = 1) and t10546 (*n* = 1). To our knowledge, this is the first report entailing baseline data on the occurrence of MRSA in RTE foods in Dhaka highlighting a potential public health risk to street food consumers.

## Introduction

*Staphylococcus aureus* (SA) is present in up to 80% of healthy individuals as a commensal, yet it is one of the most common causes of skin and soft tissue infections sometimes leading to complicated infections, such as necrotizing pneumonia, septic arthritis, endocarditis, and osteomyelitis (Popovich and Hota, 2008; David and Daum, 2010). *S. aureus* produces various toxins which are often produced in the food, and consumption of intoxicated foods potentially leads to serious diseases (Abdolshahi et al., 2018). It has many cell-associated and secreted virulence factors; some of these virulence factors include Panton-Valentine leukocidin toxin (PVL), toxic shock syndrome toxin 1 (TSST-1), hemolysins, exfoliative toxins (ETs), and staphylococcal enterotoxins (SEs) (Tong et al., 2015). PVL is a cytotoxin, related to leukocyte destruction, tissue necrosis, diffuse cellulitis, skin and soft tissue infections, necrotizing pneumonia, and osteomyelitis (Lina et al., 1999). SEs cause staphylococcal food poisoning, whereas TSST-1 and ETs are responsible for toxic shock syndrome (TSS) and staphylococcal scalded-skin syndrome (SSSS), respectively (Tong et al., 2015).

Infections caused by *S. aureus* are difficult to treat due to its ability to acquire and develop resistance to multiple antibiotics. Over the past decades, the epidemiology of methicillin-resistant *Staphylococcus aureus* (MRSA) has changed significantly. MRSA has recently been listed as one of the high-priority antibiotic-resistant pathogens by the World Health Organization (Tacconelli et al., 2017). A majority of MRSA associated with disease in hospitalized patients is known as hospital-associated (HA)-MRSA. In the early 1990s, a new type of genetically different MRSA strains has been evolved in the community known as community-associated (CA)-MRSA (Otto, 2010). Because of enhanced production of varieties of toxins, these CA-MRSA strains are exceptionally pathogenic (Cameron et al., 2011; Otto, 2012) compared to HA-MRSA. Furthermore, MRSA infections in the community caused by strains primarily associated with livestock is known as livestock-associated (LA)-MRSA (Nemati et al., 2008).

Apart from direct transmission to humans from animals, the latter being considered as a natural reservoir of this organism, transmission of MRSA might occur via exposure to or ingestion of contaminated foods. People having frequent contact with animal reservoirs or food contaminated with MRSA can become colonized with this organism and spread to the community. Food sampling and testing should be focused on foods of animal origin and especially the ready-to-eat (RTE) foods which require frequent manual handling for preparation and serving.

In Bangladesh, information on the prevalence of MRSA is currently scarce. Only a few surveys have been done in health care settings. One study among diabetic patients reported that around 37% of hospitalized and 22% of non-hospitalized patients were infected with MRSA (Jinnah et al., 1998). In a more recent study, the fraction of MRSA in hospitals of different cities in Bangladesh was shown to be 32–63%, which is much higher than in the United States and in European countries (Haq et al., 2005). There is substantial lack of information on the prevalence of MRSA in food sources in Bangladesh. Such information is useful for better understanding of the risk of exposure to MRSA through food, particularly the RTE foods.

In this study, we determined the occurrence of *S. aureus* and MRSA in retail food samples collected from local restaurants, superstores, and street vendors in Dhaka and characterized the isolates for antibiotic resistance, toxin genes, and genetic diversity using MLST and *spa* typing.

## Materials and Methods

### Food Sample Collection

Between 2010 and 2013, a total of 162 retail food samples including 112 RTE foods and 40 processed raw meat and fish products were collected from different locations in Dhaka city (Table 1). At least 100g of each sample was bought from the vendors and collected in a sterile plastic bag. Samples were kept in an ice box (+4 to 8°C) immediately after collection and transported to the laboratory within 3–4 h.

**Table 1:** Prevalence of *S. aureus* and MRSA in ready-to-eat (RTE) food and processed raw meat, fish, milk samples in Dhaka, Bangladesh.

Types of samples	No. of samples tested from each type of food	No. (%) of sample positive for *S. aureus*	No. (%) of samples positive for MRSA
Raw meat and meat products	35	6 (17.1)	1 (2.9)
Raw processed fish	5	1 (20)	1 (20)
Ready-to-eat street vended foods	112	26 (23.2)	7 (6.3)
Raw and pasteurized milk	10	2 (20)	0 (0)
Total	162	35	9


### Sample Processing

Twenty-five grams or ml (for liquid) of food sample were mixed with 225 ml of peptone saline water and homogenized. Diluted samples were spread on the Baird-Parker agar (BP) (Oxoid Ltd., Basingstoke, United Kingdom) and incubated at 37°C for 24 to 48 h. After incubation, a maximum of 3 colonies showing typical characteristics of *S. aureus* (black/dark gray with lethicinase zone) were picked up and confirmed according to the procedure described earlier (International Organization for Standardization, 1999). All coagulase positive presumptive *S. aureus* isolates were confirmed with the API STAPH system (bioMérieux S.A., France) according to manufacturer’s instructions.

### Antimicrobial Susceptibility Test

Susceptibility to antimicrobials was determined by an agar diffusion test using commercially available antibiotic disks (Oxoid Ltd., Basingstoke, United Kingdom) as described by the Clinical Laboratory Standards Institute (CLSI) guidelines (CLSI, 2012). The antimicrobial agents used were cefoxitin, chloramphenicol, ciprofloxacin, trimethoprim-sulfamethoxazole, gentamicin, tetracycline, imipenem, erythromycin, amoxicillin-clavulanic acid, and oxacillin. Isolates that showed resistance to oxacillin in disk diffusion were tested for the minimum inhibitory concentration (MIC) for oxacillin by broth dilution method described by CLSI (2012). All MRSA isolates were tested for the MIC of vancomycin by *E*-test (bioMérieux S.A., France).

### Polymerase Chain Reaction Assays for Virulence Genes

All *S. aureus* isolates were tested for a panel of virulence and pathogenic genes including the *S. aureus* thermonuclease gene (*nuc*) (Brakstad et al., 1992), Panton-Valentine leukocidin toxin gene (*pvl)* (Lina et al., 1999), staphylococcal enterotoxin genes (*sea, seb, sec, sed* and *see*) (Sharma et al., 2000), TSS toxin-1 (*tsst*) gene, exfoliative toxin genes (*eta* and *etb*) and methicillin resistance gene (*mecA*) (Mehrotra et al., 2000). DNA was extracted from bacterial isolates according to the procedure described earlier (Bollet et al., 1955).

### MLST and *Spa* Typing

All MRSA isolates were characterized by multi locus sequence typing (MLST) according to the procedure described earlier (Enright et al., 2000). Sequence types (ST) were assigned according to the MLST database^1^.

For *S. aureus* protein A (*spa*) typing, the polymorphic X region of the *spa* gene (*spa*) was amplified by PCR using the primers 1095F and 1517R according to the procedure described earlier (Harmsen et al., 2003). *spa* types were assigned by using Ridom StaphType 1.4.1 software (Ridom GmbH, Würzburg, Germany ^2^).

## Results

### Occurrence of *S. aureus* in Food Samples

Of the 162 samples, 35 (22%) were positive for *S. aureus*. Among these, 26 isolates were isolated from RTE foods and 9 from raw processed foods.

### Antibiotic Susceptibility of *S. aureus*

Antibiotic susceptibility test of the *S. aureus* isolates showed that 74% of isolates were resistant to erythromycin, 49% to ciprofloxacin, 31% to oxacillin, 26% to cefoxitin, 20% to amoxicillin-clavulanic acid, 20% to tetracycline, 11% to trimethoprim-sulfamethoxazole, 6% to imipenem and 3% to gentamicin. None of the isolates were resistant to chloramphenicol (Table 2). Around 37% (*n* = 13) of isolates were multidrug resistant (MDR) (resistant to 3 or more classes of antibiotics). MIC for oxacillin was found ≥8 μg/ml for isolates that were identified as resistant in disk diffusion method. All MRSA isolates were found to be sensitive to vancomycin.

**Table 2:** Antimicrobial resistance of *S. aureus* strains isolated from RTE food and raw food samples (processed raw meat, fish and milk) in Dhaka, Bangladesh.

Antimicrobial Agents	Resistant Breakpoint (zone of diameter in mm) (CLSI, 2012)	No. (%) of resistant *S. aureus* isolates		
		Ready-to-eat foods (*n* = 26)	Raw foods (*n* = 9)	Total isolates (*n* = 35)
Erythromycin	≤13	20 (77)	6 (66.7)	26 (74.3)
Ciprofloxacin	≤15	12 (46.2)	5 (55.6)	17 (48.6)
Oxacillin	≤10	7 (27)	4 (44.4)	11 (31.4)
Cefoxitin	≤21	6 (23.1)	3 (33.3)	9 (25.7)
Amoxicillin-clavulanic acid	≤19	4 (15.4)	3 (33.3)	7 (20)
Tetracycline	≤14	5 (19.2)	2 (22.2)	7 (20)
Trimethoprim- sulfamethoxazole	≤10	4 (15.4)	0 (0)	4 (11.4)
Imipenem	≤13	2 (7.7)	0 (0)	2 (5.7)
Gentamicin	≤12	1 (3.8)	0 (0)	1 (2.9)
Chloramphenicol	≤12	0 (0)	0 (0)	0 (0)


### Toxigenic Characteristics of *S. aureus*

**FIGURE 1:**
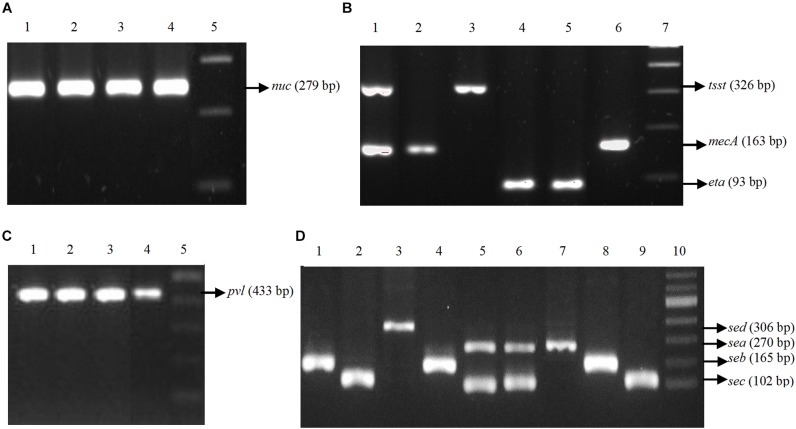
Agarose gel electrophoresis of PCR amplified genes present in the *Staphylococcus aureus* isolated from RTE foods. The individual gene product was characterized by comparison with standard molecular size marker. **(A)** PCR for *nuc* gene. Lane 1–3: Samples, Lane 4: Positive Control (PC), Lane 5: 100 bp DNA ladder. **(B)** PCR for *mecA, tsst-1 and eta* genes. Lane 1: PC for *mecA* and *tsst-1*, Lane 2–4: Samples, Lane 5: PC, Lane 6: Sample, Lane 7: 100 bp DNA ladder. **(C)** PCR for *pvl* gene. Lane 1–3: Samples, Lane 4: PC, Lane 5: 100 bp ladder. **(D)** PCR for enterotoxin genes. Lane 1–6: Samples, Lane 7: PC for *sea*, Lane 8: PC for *seb*, Lane 9: PC for *sec*, Lane 10: 100 bp DNA ladder.

All *S. aureus* isolates were positive for thermonuclease gene (*nuc*). About 71% of isolates (*n* = 25) were positive for *pvl* gene. More than 74% of isolates (*n* = 26) were positive for enterotoxin genes (*sea* = 26%, *n* = 9; *seb* = 11%, *n* = 4; *sec* = 49%, *n* = 17 and *sed* = 3%, *n* = 1) (Table 3). In each case, a PCR product of the expected size was generated (Figure 1). The frequencies of other genes are listed in Table 3. Nine (26%) isolates were positive for *mecA* gene either alone (2.8%, *n* = 1) or in tandem with other genes (*sec-mecA-tsst1-pvl, seb-mecA, sea-mecA, sea-mecA-pvl, seb-mecA-pvl*). None of the isolates were positive for *see* and *etb* genes.

**Table 3:** Prevalence of different toxin genes in *S. aureus* isolates from RTE food and raw food samples (processed raw meat, fish and milk) in Dhaka, Bangladesh.

Sources of strain	No. (%) of strains positive for:									
	*pvl*	*se*					*tsst-1*	*et*		*mecA*
		*a*	*b*	*c*	*d*	*e*		*a*	*b*	
Ready-to-eat foods (*n* = 26)	21 (84)	6 (66.7)	3 (75)	14 (82.3)	0 (0)	0 (0)	5 (83.3)	4 (100)	0 (0)	8 (88.9)
Raw foods ( *n* = 9)	4 (16)	3 (33.3)	1 (25)	3 (17.6)	1 (100)	0 (0)	1 (16.7)	0 (0)	0 (0)	1 (11.1)
Total ( *n* = 35)	25 (71.4)	9 (25.7)	4 (11.4)	17 (48.6)	1 (2.9)	0 (0)	6 (17.1)	4 (11.4)	0 (0)	9 (25.7)


### Identification and Characterization of MRSA

Of the 35 *S. aureus* isolates, 9 (26%) were detected as MRSA, which represents 6% of total number of food samples (*n* = 162) tested in the study. Of these 9 isolates, 6 were isolated from RTE foods mostly served in the road side small restaurants and street vendors, 2 from processed raw meat samples and 1 from processed fish sample. All but one MRSA isolates were resistant to both oxacillin and cefoxitin, with an MIC of oxacillin ≥16 μg/ml. All MRSA isolates were MDR. Of the 9 MRSA isolates, 4 (44%) were positive for *pvl* gene, 3 isolates of each were positive for *sea* and *seb* genes, respectively and 2 isolates were positive for *sec* gene. Isolates positive for *pvl* gene were positive for at least one additional enterotoxin gene (Table 4).

**Table 4:** Characteristics of MRSA isolated from RTE food and raw food samples (processed raw meat, fish and milk) in Dhaka, Bangladesh.

Category of sample	Type of Sample	Toxin gene profile								Antibiotic resistance pattern^a^	MIC value of oxacillin (≤2 = S, ≥4 = R)	MLST	*spa* typing
		*sea*	*seb*	*sec*	*mecA*	*tsst-1*	*eta*	*etb*	*pvl*				
Processed raw fish	Fish finger	–	–	+	+	+	–	–	–	AMC, E, TE, CIP, OX, FOX	16 μg/ml (R)	ST 361	t315
Processed raw meat	Meat ball	–	+	–	+	–	–	–	–	AMC, E, OX, FOX	16 μg/ml (R)	ST 80	t8731
	Beef kebab	+	–	–	+	–	–	–	–	AMC, E, OX, FOX	32 μg/ml (R)	ST 6	t304
Ready-to-eat foods	Burger	–	+	–	+	–	–	–	+	AMC, E, OX, FOX	32 μg/ml (R)	ST 80	t1198
	Pastry	–	–	–	+	–	–	–	–	AMC, E, TE, IPM, CIP, OX, FOX	≥128 μg/ml (R)	ST239	t275
	Chatpati	–	–	+	+	+	–	–	+	E, TE, CIP, OX, FOX	32 μg/ml (R)	ST 361	t315
	Salad	+	–	–	+	–	–	–	+	E, OX	16 μg/ml (R)	ST 6	t10546
	Fuska	+	–	–	+	–	–	–	–	AMC, E, TE, IPM, CIP, CN, OX, FOX, SXT	≥128 μg/ml (R)	ST239	t037
	Sweet	–	+	–	+	–	–	–	+	AMC, E, OX, FOX	64 μg/ml (R)	ST 80	t1198


*^a^AMC, Amoxicillin-clavulanic acid; E, Erythromycin; TE, Tetracycline; CIP, Ciprofloxacin; OX, Oxacillin; FOX, Cefoxitin; IPM, Imipenem; CN, Gentamicin; SXT, Trimethoprim-sulfamethoxazole*.

### Genotyping of MRSA

A total of 4 sequence types (ST) were identified among 9 MRSA isolates of which 3 isolates belonged to ST80 and 2 isolates in each belonged to ST6, ST239 and ST361. A total of 7 different *spa* types were detected among 9 MRSA isolates, of which t1198 and t315 were the predominant one (2 isolates in each type), followed by t8731, t304, t275, t10546, t037 (1 isolate in each type).

## Discussion

Foodborne transmission of MRSA is a global concern and therefore the prevalence and genetic characteristics of these organisms need to be thoroughly studied. This study provides the first evidence of the occurrence of MRSA in RTE food in Bangladesh. Around 23% (26/112) of RTE food samples collected from Dhaka city were found positive for *S. aureus* and 5% (6/112) were identified as MRSA. This rate is relatively higher than the reports from other countries, for example, the prevalence of MRSA in dairy products from Italy was 0.5% (Carfora et al., 2015) and 1.3% in retail foods from China (Yang et al., 2016).

Contamination of RTE foods with *S. aureus* can easily occur due to poor hygienic practices of food handlers during food preparation as it is known that 50–70% of healthy individuals serve as carriers of *S. aureus* (Solberg, 2000; Le Loir et al., 2003). Like many other resource poor settings, street foods in Dhaka city are often processed and served with bare hands. Although there is no data on the proportion of street food vendors in Dhaka city have *S. aureus* on their hands but a study in neighboring country India showed that 36% of hand rinse samples (*n* = 83) collected from workers responsible for food preparation, serving and cleaning, carried oxacillin resistant *S*. *aureus* (Kasturwar and Shafee, 2011). A similar study in Zimbabwe showed that 32% of food handlers carried *S. aureus* on their hands, while only 6.4% carried *E. coli* (Gadaga et al., 2008).

Clinical management of Staphylococcal infection is relied on antibiotic treatment which often fails due to aggressive resistance of organisms to antibiotics. We found that a high proportion of isolates in this study were resistant to erythromycin (74%) and ciprofloxacin (49%) while none of the isolates was resistant to chloramphenicol. It indicates that this first generation antibiotic may serve as an alternative to the newer generation of more expensive antibiotics in resource poor settings, if infections are caused by these organisms.

Characterization of foodborne bacterial isolates for pathogenic properties provides important information on the ability of the isolates to cause human infection. We tested all *S. aureus* isolates for different pathogenic genes. We found that *pvl* was present in 71% of all *S. aureus* and in 44% of MRSA isolates. *pvl* is an important virulence gene of *S. aureus*, which is mainly found in clinical MRSA isolates, predominantly associated with community associated infections (CA-MRSA) (Pu et al., 2009; Hanson et al., 2011; Wang et al., 2014). The *pvl* gene is also considered to be a stable genetic marker for CA-MRSA (Deurenberg et al., 2007). The presence of *pvl* in large number of isolates in this study indicates the possible contamination of food via human sources and consequently contaminated food can serve as a source of CA-MRSA. Among classical enterotoxin genes, *sec* gene was predominantly found in *S. aureus* isolates (37%) while in case of MRSA isolates, *sea* and *seb* were more common (Tables 3, 4). Epidemiological studies indicate that the majority of *S. aureus* infections and outbreaks have been caused by isolates with SEA type toxins, followed by isolates with SED, SEC and SEB toxin types (Asao et al., 2003; Ikeda et al., 2005; Cha et al., 2006; Kerouanton et al., 2007; Argudin et al., 2010). Among other toxin genes, *tsst-1* (toxic shock syndrome toxin 1) and *eta* (an enterotoxin) were found in 17 and 9% of the *S. aureus* isolates, respectively. Although these toxins are mostly associated with human isolates, there are sporadic reports on the prevalence of *S. aureus* carrying these toxin genes from food sources (Hammad et al., 2012; Yang et al., 2018). Interestingly, one isolate was positive for multiple virulence genes including *sec*, *tsst-1*, *eta* and *pvl* indicating the potential ability of this isolate to cause human infection.

The genetic types of all MRSA isolates were characterized by MLST and *spa* typing. Of the 9 MRSA isolates, 3 belonged to the Sequence Type 80, two of these were *pvl* positive, had the same *spa* type (t1198) both isolated from RTE foods but of different types and from different locations (Table 4). *pvl* positive ST80 is predominantly found among CA-MRSA isolates in Europe and Middle East and they were associated with severe skin/soft tissue infections and necrotizing pneumonia (Budimir et al., 2010). The other genotypes found among MRSA isolates in this study were ST239-t037/t275, ST6-t304/t10546 and ST361-t315 (*n* = 2). All these genotypes were previously reported from clinical isolates of MRSA obtained from hospitalized patients. For example, the ST239-t037 was reported as the most common genotype among hospitalized burn patients in Iran and from hospitalized patients with wound/soft tissue infections and respiratory infections in Malaysia (Ghaznavi-Rad et al., 2010; Goudarzi et al., 2017). ST6-t304 was reported as the predominant genotype isolated from patients with wound/soft tissue infections at a tertiary hospital in the Sultanate of Oman (Udo et al., 2014). ST239-t037 and ST6-t304 clones of CA MRSA reported from Iran and Malaysia were *pvl* negative and a majority of ST239-t037 was positive for *sea* gene, which is similar to the characteristics of food isolates of the same genetic types found in this study (Table 4).

In conclusion, we report the first investigation of *S. aureus* from retail, RTE foods in Dhaka, Bangladesh. The contamination of *S. aureus* was common in RTE foods with a high prevalence of MRSA. All MRSA isolates were resistant to multiple antibiotics and a majority of these were positive for more than one toxin gene indicating their pathogenic potential. Genetic types of MRSA isolates in this study matched with the epidemic and pandemic clones of CA-MRSA. Our findings therefore strongly hint at the potential role of contaminated foods in the dissemination of multi-drug resistant *S. aureus* strains. A systematic surveillance of MRSA coupled with a focused educational and awareness campaign should be undertaken along the entire food production and supply chain, especially targeting the sectors involved with RTE foods. Furthermore, the findings described herein could also be generally relevant to the developing country settings of Asia, Africa and all other places where RTE food is sold and consumed.

## Author Contributions

MI and SP developed the project and designed the research. MR, MH, AN, ZK, and JW performed the experiments. MI, MR, and MH wrote the manuscript. All authors analyzed and discussed the data, contributed to the writing of the statement and agreed with its content and conclusions, and read and approved the final manuscript.

## Conflict of Interest Statement

The authors declare that the research was conducted in the absence of any commercial or financial relationships that could be construed as a potential conflict of interest.

## Footnotes

1 http://www.mlst.net/

2 www.spaserver.ridom.de
